# A SCOPING REVIEW FOR PERSON-FOCUSED FALL PREVENTIVE INTERVENTIONS IN LONG-TERM CARE FACILITIES

**DOI:** 10.1093/geroni/igad104.2270

**Published:** 2023-12-21

**Authors:** Wonkyung Jung, Sungwon Lim, Hilaire Thompson

**Affiliations:** Johns Hopkins University, Baltimore, Maryland, United States; University of Washington, Seattle, Washington, United States; University of Washington, Seattle, Washington, United States

## Abstract

Falls are a significant concern in long-term care facilities (LTCFs) as fall-related injuries (FRI) can result in functional impairment, disability, and death. Older adults living in LTCFs are at greater risk for falls than those in the community. Using scoping review methodology, we aimed to synthesize evidence examining intervention effects of person-focused interventions for risk assessment and prevention in LTCFs to identify evidence-based practices. Databases searched included Medline, CINAHL, and EMBASE to identify original research from 2017 to April 2022 in accordance with the Preferred Reporting Items for Systematic reviews and Meta-Analyses extension for Scoping Reviews (PRISMA-ScR) guideline. Following the removal of duplicates, 888 articles were identified for possible review, following title and abstract screening 84 studies underwent full-text review against inclusion/exclusion criteria, with 30 studies remaining for analysis. We conducted a narrative synthesis to summarize the included studies. Evidence-based interventions for fall prevention identified include (1) exercise programs (e.g., high-intensity functional exercise, aerobic exercise, short stick exercises, etc.); (2) balance training programs (e.g., Wii Fit); (3) multifaceted, nurse-led education; and (4) others (e.g., whole body vibration, and lavender olfactory stimulation). Outcomes of included studies included the number of falls, fall rate, risk of falls, and fear of falls before and after interventions. Overall, most studies reported significant effects of person-focused interventions in LTCFs. Available evidence supports that well-designed person-focused interventions can reduce falls and fear of falls for individuals in LTCFs.

